# The Influence of Felt Trust on Nurses' Job Performance: The Mediating Role of Self-Efficacy and Moderating Role of Benevolent Leadership

**DOI:** 10.1155/jonm/8462843

**Published:** 2025-10-22

**Authors:** Xiaolin Shen, Tao Shen, Diwei Zheng, Ying Li

**Affiliations:** ^1^Department of Rheumatology and Immunology, West China Hospital, Sichuan University, 37 Guoxue Ave., Wuhou District, Chengdu 610030, Sichuan, China; ^2^School of Business Administration, Faculty of Business Administration, Southwestern University of Finance and Economics, 555 Liutai Ave., Wenjiang District, Chengdu 610030, Sichuan, China; ^3^School of Management, Xiamen University, Siming South Road 422, Xiamen 361005, Fujian, China

**Keywords:** benevolent leadership, felt trust, job performance, nurses, self-efficacy

## Abstract

**Background:**

The concept of felt trust (FT)—nurses' perception of being trusted by their supervisors—is increasingly recognized as integral to the quality of healthcare delivery. Despite its importance, the specific impact of FT on nursing performance and the role of self-efficacy and benevolent leadership (BL) in this context are not well understood.

**Objective:**

This study aims to explore the influence of nurses' FT on their job performance and the mediating role of self-efficacy and moderating role of BL in this relationship.

**Design:**

A cross-sectional design was adopted to survey 320 registered nurses from 15 public hospitals in China. Data were collected from March to April 2022.

**Methods:**

The study utilized hierarchical regression analysis and the bootstrap method in the PROCESS macro to test the proposed hypotheses.

**Results:**

The findings indicate that FT has a positive and significant effect on job performance among nurses (*β* = 0.54, *p* < 0.01). Moreover, self-efficacy mediates the relationship between FT and nurses' job performance (indirect effect = 0.41, 95% CI [0.33, 0.50]). Furthermore, nurses' perceived BL moderates the relationship between FT and self-efficacy, with a stronger positive effect observed among nurses with high levels of BL (*β* = 0.07, *p* < 0.01).

**Conclusion:**

This study suggests that FT is an important factor that influences job performance among nurses. Self-efficacy plays a significant mediating role in this relationship, while BL has a moderating effect on the relationship between FT and self-efficacy. These findings highlight the importance of promoting FT and self-efficacy among nurses while also cultivating a supportive organizational culture that fosters BL.

**Implications for Nursing Management:**

This study highlights the importance of fostering FT to enhance nurses' job performance. Nursing management can benefit from creating an environment that promotes FT and self-efficacy and encourages BL, leading to improved patient care and nurse satisfaction.

## 1. Introduction

Nurses' job performance is critical for ensuring quality patient care, achieving high levels of patient satisfaction, and maintaining the success and reputation of healthcare organizations [1, 2]. A substantial body of research has explored the influence of various factors, such as leadership styles, workload, and work alienation, on nurses' job performance [2, 3, 4]. However, limited attention has been given to the concept of felt trust (FT), which refers to an individual's perception of being trusted by another [5, 6]. Within the context of organizational management, trust often manifests as employees' perceptions of reliance and openness exhibited by their supervisors, commonly described as FT. For instance, delegating significant responsibilities or sharing sensitive information with subordinates may signal trust. When a supervisor entrusts an employee with a critical task, it may be interpreted as an affirmation of trust, thereby fostering a sense of FT. Prior research has established FT as an important predictor of job performance across various domains [7]. Nevertheless, the role of nurses' FT from their head nurses in shaping job performance remains underexplored in nursing management literature.

To bridge this gap, this study investigates the impact of FT on nurses' job performance in healthcare organizations. FT, as a subjective perception, can profoundly influence employees' attitudes and behaviors in the workplace [6]. In the nursing context, nurses who perceive trust from their head nurses may feel valued and experience an enhanced sense of self-worth, motivating them to meet leadership expectations. Research rooted in self-consistency theory suggests that individuals strive to maintain their self-concepts by aligning behaviors with their perceived role expectations [8]. As a result, nurses with high levels of FT are likely to be more motivated to fulfill their responsibilities effectively, exert extra effort, and achieve superior job performance [9]. Therefore, it is hypothesized that FT positively influences nurses' job performance.

This study also seeks to uncover the underlying mechanisms of the relationship between FT and job performance by examining the mediating role of self-efficacy and the moderating role of benevolent leadership (BL). Specifically, we propose that self-efficacy—a nurse's belief in their ability to successfully complete job-related tasks [10]—mediates this relationship. Trust from head nurses can serve as both a compliment and an indication of confidence in nurses' abilities, fostering psychological empowerment and enhancing feelings of pride. These factors, in turn, may boost self-efficacy, leading to improved job performance. Accordingly, it is expected that self-efficacy mediates the link between FT and job performance.

Furthermore, this study posits that BL—characterized by leaders' genuine concern for their subordinates' well-being and success [11, 12]—moderates the relationship between FT and self-efficacy. We hypothesize that the positive effect of FT on self-efficacy is amplified under conditions of high BL compared to low BL.

**Figure 1:**
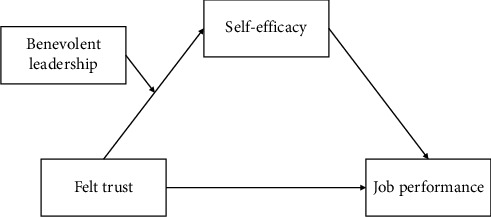
The conceptual model of this study.

In summary, this study aims to explore the influence of nurses' FT on job performance, as well as the mediating role of self-efficacy and the moderating role of BL. The conceptual model of this research is presented in Figure 1. By identifying FT as a critical antecedent to improved job performance and elucidating the mechanisms underlying this relationship, this study contributes to the nursing management literature. The findings offer practical insights for healthcare organizations striving to enhance nurses' job performance and, ultimately, improve patient outcomes.

## 2. Theoretical Background and Hypothesis

### 2.1. FT and Job Performance of Nurses

The term “FT” refers to an individual's subjective perception of the trust others place in them [6]. In the context of nursing, FT can be defined as the extent to which nurses perceive trust from their head nurses. For example, FT is demonstrated when head nurses exhibit unwavering confidence in a nurse's ability to perform their responsibilities effectively. Conversely, nurses' job performance refers to the degree to which their actions and behaviors align with the objectives and goals of their healthcare organization.

Trust is a cornerstone of social exchange theory, which posits that social interactions shape individuals' behaviors and responses toward one another [6]. According to this theory, individuals who perceive trust from others are inclined to reciprocate with positive actions and behaviors. Within the healthcare setting, trust fosters effective communication, collaboration, and teamwork among professionals [13]. Nurses who feel trusted by their supervisors are likely to experience a sense of obligation and commitment to reciprocate this trust [7]. Consequently, these nurses may exhibit behaviors that advance organizational goals, such as exceeding job expectations and demonstrating strong organizational commitment. Furthermore, FT has the potential to improve nurses' job satisfaction and overall well-being, which can further enhance their job performance [14]. Drawing on social exchange theory and existing research on the relationship between trust and job performance, it is reasonable to propose that higher levels of FT are positively associated with improved job performance among nurses.

**Hypothesis 1.**

FT is positively related to the job performance of nurses.

### 2.2. The Mediating Role of Self-Efficacy

This study posits that self-efficacy mediates the relationship between FT and nurses' job performance. According to social cognitive theory [15], individuals' beliefs about their capabilities to perform specific tasks or behaviors—referred to as self-efficacy—significantly influence their motivation, behavior, and performance. Therefore, it is plausible that self-efficacy plays a crucial role in linking FT to nurses' job performance.

FT fosters a supportive work environment that encourages collaboration, open communication, and teamwork. Nurses who perceive trust from their head nurses are more likely to engage in honest and transparent communication, offer assistance to colleagues, and work collaboratively. Such teamwork and collaboration can enhance their self-efficacy. Trust inherently involves the trustor's expectation of certain future behaviors from the trustee. Thus, when leaders make followers feel trusted, they convey high expectations for them. When followers perceive these high expectations, it can become a self-fulfilling prophecy, enhancing their self-efficacy, motivation, and behavior. This phenomenon, known as the Pygmalion effect [9], suggests that followers who feel a high level of trust are likely to have greater confidence in their ability to perform their job, resulting in increased self-efficacy.

Nurses with high self-efficacy are more inclined to undertake challenging tasks and responsibilities, which can enhance their job performance. These nurses tend to be more confident in managing complex patient care and responding to emergencies, leading to increased problem-solving and critical thinking activities that boost their job performance. Furthermore, high self-efficacy is essential for nursing professionals who frequently encounter significant stress and uncertainty. The ability to maintain a positive outlook and handle difficult situations with confidence can markedly influence their job performance. Research in social psychology and organizational studies has uncovered evidence that an increase in self-efficacy can lead to greater effort and persistence in completing tasks, ultimately resulting in improved performance [16]. These findings have been supported by empirical studies examining both in-role and extra-role job performance. In conclusion, based on social cognitive theory and previous research, we anticipate that self-efficacy mediates the relationship between FT and nurses' job performance.

**Hypothesis 2.**

Self-efficacy mediates the relationship between FT and job performance of nurses.

### 2.3. The Moderating Role of BL

BL is widely recognized as a leadership style that emphasizes prioritizing the well-being and personal growth of employees [17]. This leadership approach has gained particular importance in the healthcare sector, where supportive and empathetic leadership behaviors have been shown to significantly improve nurses' job satisfaction, mental well-being, and overall performance [18]. By exploring the role of BL in enhancing the relationship between FT and self-efficacy, nursing management can derive actionable insights for developing effective leadership strategies. These strategies may include targeted leadership training programs and interventions designed to promote trust, bolster self-efficacy, and ultimately enhance job performance among nurses, benefiting both individuals and organizations.

When nurses perceive that their head nurses demonstrate a strong commitment to their well-being and personal development, the positive impact of FT on self-efficacy is likely to be strengthened. Benevolent leaders exhibit genuine care and concern for their employees by providing necessary resources, guidance, and emotional support, all of which are essential for cultivating self-efficacy. Such leadership behaviors not only enhance trust but also create an empowering work environment where nurses feel confident in their abilities to overcome challenges. Prior research has consistently highlighted the pivotal role of BL in fostering self-efficacy. For instance, Xia et al. [19] identified a positive association between BL and employees' creative self-efficacy, emphasizing the importance of supportive leadership in driving confidence and innovation. Similarly, Xu et al. [8] demonstrated that BL significantly contributes to the development of self-efficacy, further reinforcing its value in organizational settings.

Conversely, when nurses perceive a lack of BL, they are likely to encounter inadequate support and resources from their head nurses. For example, head nurses who do not prioritize their subordinates' career development or fail to offer sufficient guidance may leave nurses feeling undervalued and unsupported. This lack of encouragement and assistance can hinder nurses' ability to enhance their professional skills and competencies, leading to diminished confidence in their abilities and, ultimately, lower self-efficacy. In such scenarios, nurses may find it challenging to meet the demands of their roles effectively, which could negatively impact their performance and overall job satisfaction.

In conclusion, grounded in both theoretical frameworks and empirical evidence, this study proposes that BL serves as a key positive moderator in the relationship between FT and self-efficacy. By fostering an environment of support and care, BL not only enhances self-efficacy but also strengthens the trust that nurses perceive from their leaders, contributing to improved individual and organizational outcomes.

**Hypothesis 3.**

BL positively moderates the relationship between FT and self-efficacy such that this relationship will be stronger when BL is high rather than low.

## 3. Materials and Methods

### 3.1. Sample and Procedures

The objective of this study was to examine the association between FT and job performance among nurses, with a focus on the mediating effect of self-efficacy and the moderating effect of BL. The study utilized a cross-sectional design and administered a questionnaire survey to collect data from March 2022 to April 2022. The G^∗^Power estimation was used to calculate the minimum sample size required, which was determined to be 270, considering an effect size of 0.15, an error probability of 0.05, and a power of 0.8.

Convenience sampling was employed to recruit 343 nurses working in 15 tertiary and secondary public hospitals in Sichuan Province, China. The participants were required to meet specific inclusion criteria, including having at least 1 year of experience as a registered nurse, understanding the purpose of the study, and currently working in their current ward. A total of 325 questionnaires were completed, representing a response rate of 94.8%. After excluding five questionnaires due to incomplete or illogical answers, data from 320 participants were analyzed, exceeding the minimum sample size requirement of 270.

### 3.2. Measures

In this study, reliable and valid measurement instruments were utilized to assess the four main study variables, namely FT, self-efficacy, job performance, and BL. The measurement instruments were adapted from established studies that have demonstrated strong psychometric properties. Responses from the participants were collected using a five-point Likert scale, where a score of five indicated *strongly agree* and a score of one indicated *strongly disagree*. In addition to the four main study variables, demographic data were also collected from the participants. The demographic information collected included gender, age, education level, marital status, income, hospital level, years of experience working in the nursing unit, and positional rank. The measurements for the four main variables are as follows.

#### 3.2.1. FT

FT was measured using a 3-item scale adapted from Cho et al. [20]. A sample item is “My nurse leader places trust in me.” The validity and reliability coefficients of the measurement instrument in this study were satisfactory (Cronbach's *α* = 0.94, average variance extracted [AVE] = 0.78, composite reliability [CR] = 0.96).

#### 3.2.2. Self-Efficacy

The self-efficacy of nurses was assessed using an eight-item scale from Fast et al. [21]. A sample item reads, “When facing difficult tasks, I am certain that I will accomplish them.” The measurement instrument exhibited adequate reliability and validity in this study (Cronbach's *α* = 0.97, AVE = 0.83, CR = 0.97).

#### 3.2.3. Job Performance

A five-item scale from Williams and Anderson [22] was utilized to measure job performance of nurses. Questions included “I fulfill responsibilities specified in job description.” This measurement is widely used for job performance and exhibited adequate reliability and validity in various research contexts (Cronbach's *α* = 0.95, AVE = 0.82, CR = 0.96).

#### 3.2.4. BL

We measured BL using an eleven-item scale from Shaw and Liao [17]. A sample item is “My nurse leader is like a family member when he/she gets along with us.” The measurement instrument had adequate reliability and validity in this study (Cronbach's *α* = 0.97, AVE = 0.82, CR = 0.98).

### 3.3. Ethical Consideration

This study received approval from the institutional review board of the hospital affiliated with the primary author in Sichuan Province, China. Before the participants took part in the study, they were given a brief overview of the research objectives and the significance of their involvement. They were also informed about the potential risks and benefits of participating in the study. The participants were required to provide their informed consent before taking part in the study, which ensured that they fully understood the details and were willing to participate in the research. To maintain the confidentiality of the participants, all data collected during the study were stored securely and accessible only by the researchers involved in the project. The participants were not identified in any publications or presentations resulting from the study. Overall, the research was conducted with the utmost ethical consideration, and the privacy and autonomy of the participants were respected throughout the study.

### 3.4. Data Analysis

The data analysis for this study was conducted using IBM SPSS Version 26.0, a software product of IBM Inc., Armonk, NY, United States. In addition, the PROCESS Procedure Version 3.5 in SPSS was utilized to analyze the collected data. The demographic characteristics of the participants were presented using descriptive statistics. Correlations between the study variables were evaluated using Pearson's correlation coefficient. Moreover, the hypothesized relationships were tested through the application of hierarchical regression analysis along with the use of the bootstrap method. All statistical outcomes were considered statistically significant at a significance level of *p* < 0.05.

## 4. Results

### 4.1. Confirmatory Factor Analysis (CFA)

To assess the discriminant validity among four primary variables: FT, BL, self-efficacy, and job performance, CFA was employed using AMOS 22.0 software. Eight CFA models were tested, including one baseline model (four-factor model) and several alternative measurement models (six three-factor models and a one-factor model). To evaluate the discriminant validity among the variables, the fit indicators of the baseline model and alternative measurement models were compared based on Cheung and Rensvold's [23] recommendations. As shown in Table 1, the fit indicators of the four-factor model (*χ*^2^ = 779.04, df = 301, NC (*χ*^2^/df) = 2.59, IFI = 0.97, CFI = 0.96, RMSEA = 0.07) were significantly better than those of the alternative models. These results indicate that the four primary variables of the current study, FT, BL, self-efficacy, and job performance, exhibit good discriminant validity in terms of both connotation and measurement and can be regarded as distinct constructs.

### 4.2. Descriptive Statistics

The questionnaire was completed by a sample of 320 nurses.  Table 2 presents the demographic and job-related characteristics of the participants. As depicted in Table 2, the overwhelming majority of the participants were female (94.7%). Furthermore, more than 90% of the participants were under the age of 40, with 146 participants (45.6%) under the age of 30 and 152 participants (47.5%) aged between 31 and 40. Over 65% of the participants reported an annual income between 50,000 and 150,000 RMB, with 103 participants (32.2%) having an annual income between 50,000 and 100,000 RMB and 110 participants (34.4%) having an annual income between 110,000 and 150,000 RMB. Additionally, more than 95% of the participants had less than 20 years of work experience, with 116 participants (36.3%) having 6–10 years of work experience. Furthermore, 252 participants (78.8%) held a bachelor's degree, 257 participants (80.3%) came from Grade IIIA hospitals, and 192 participants (60%) held the title of nurse practitioner.

### 4.3. Correlation Analysis

Table 3 displays the means, standard deviations, and correlation coefficients of all variables in this study. The score range indicates that all primary variables are at high levels: FT (*M* = 4.01, SD = 0.80), BL (*M* = 3.82, SD = 0.86), self-efficacy (*M* = 4.11, SD = 0.66), and job performance (*M* = 4.22, SD = 0.62). The results of Pearson's correlation analysis revealed significant positive correlations between FT and self-efficacy (*r* = 0.75, *p* < 0.01), FT and job performance (*r* = 0.71, *p* < 0.01), and self-efficacy and job performance (*r* = 0.85, *p* < 0.01). Moreover, there were also significant positive correlations between BL and other primary variables. These findings provide preliminary evidence for subsequent hypothesis testing, indicating that the primary variables in this study are positively associated with each other.

### 4.4. Hypothesis Test

#### 4.4.1. Main Effect and Mediating Effect

To test the hypotheses of this study, hierarchical regression analysis was employed, and the results are presented in Table 4. The results indicated that FT was significantly and positively related to job performance (*β*_1_ = 0.54, *p* < 0.01, M4), thus supporting Hypothesis 1.

Hypothesis 2 proposed that self-efficacy mediates the relationship between FT and job performance. As shown in Table 4, using Baron and Kenny's [24] three-step regression method, we confirmed the direct effect of FT on job performance (*β*_1_ = 0.54, *p < *0.01), followed by its significant influence on self-efficacy (*β*_2_ = 0.62, *p* < 0.01, M1). When introducing self-efficacy into the model, the effect of FT on job performance was attenuated but remained significant (*β*_1_′ = 0.13, *p* < 0.01, M6), while self-efficacy robustly predicted job performance (*β*_3_′ = 0.66, *p* < 0.01, M6). Therefore, self-efficacy played a partially mediating role between FT and job performance, supporting Hypothesis 2.

**Figure 2:**
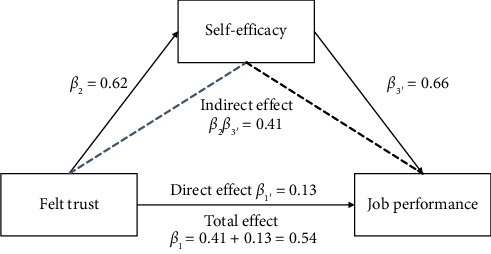
Mediating effect of self-efficacy.

To further test the mediating role of self-efficacy, the SPSS PROCESS Macro Model 4 [25] was utilized. The results of the bootstrap method test with 5000 bootstrap samples are presented in Table 5, indicating that the indirect effect of FT on job performance through self-efficacy was significant (*β*_2_*β*_3_′ = 0.41, *p* < 0.01), and the 95% bias-corrected confidence interval (CI) of this effect [0.33, 0.50] excluded 0, thereby providing further support for Hypothesis 2. The effect sizes of the main, direct, and indirect effects of the model in this study are illustrated in Figure 2.

#### 4.4.2. Moderating Effect

To examine the moderating effect of BL on the relationship between FT and self-efficacy, hierarchical regression analysis was conducted in this study. Before the analysis, the independent variable (FT) and the moderating variable (BL) were standardized to avoid potential multicollinearity, and an interaction term (FT × BL) was created by multiplying their standardized values. The results presented in Table 4 indicated that the interaction effect between FT and BL was positively and significantly related to self-efficacy (*β* = 0.07, *p* < 0.01, M4). This finding suggests that the positive effect of FT on self-efficacy is stronger when BL is high, supporting Hypothesis 3.

**Figure 3:**
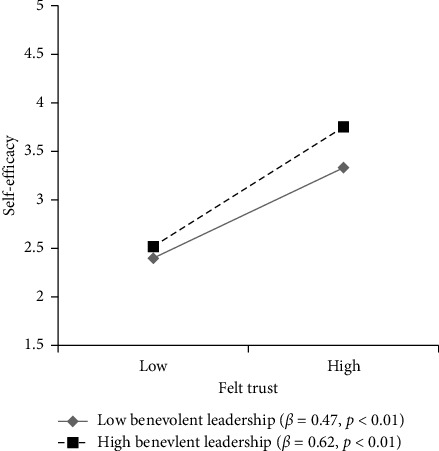
Moderating effect of benevolent leadership.

To further illustrate the nature of the moderating effect, a plot of the interaction effect was generated following the suggestion of Cohen et al. [26]; using one standard deviation above and below the mean of BL (see Figure 3). As illustrated in Figure 3, when BL was high (mean + SD), the positive correlation between FT and self-efficacy was stronger (*β* = 0.62, *p* < 0.01), while when BL was low (mean − SD), the positive correlation between FT and self-efficacy weakened (*β* = 0.47, *p* < 0.01). This result is consistent with the prediction of Hypothesis 3.

## 5. Discussion

This study contributes to the understanding of key dynamics within the nursing profession by exploring the influence of FT on job performance, along with the pivotal roles of self-efficacy and BL. Our findings align with a robust body of literature that underscores the critical role of trust in fostering positive workplace outcomes, particularly in the demanding and high-stakes environment of healthcare [27, 28, 29, 30]. The mediating role of self-efficacy identified in this research resonates with Bandura's [31, 32] seminal work, which highlights self-efficacy as a central determinant of behavioral change and performance outcomes. These results are further corroborated by empirical studies demonstrating the predictive strength of self-efficacy on job performance across various professional domains, including nursing [16, 33, 34, 35].

This study also adds to the growing body of research on BL, a leadership style that has been consistently linked to positive organizational outcomes, such as enhanced creativity, job satisfaction, and job performance [19, 36]. Our findings regarding the moderating effect of BL on the relationship between FT and self-efficacy align with recent evidence suggesting that supportive leadership styles can amplify the beneficial impacts of trust on employee outcomes [37, 38]. BL provides a nurturing environment that reinforces employees' confidence, fostering a sense of empowerment and personal growth.

By situating this research within a Chinese cultural context, the study addresses a critical gap in the existing literature, which predominantly focuses on Western settings. Expanding the geographical scope of this research is both timely and essential for capturing the cultural nuances that influence the relationships between FT, self-efficacy, BL, and job performance [9]. Our findings offer unique insights that broaden the understanding of these constructs, thereby contributing to the global discourse on nursing practices and leadership.

Theoretically, this study integrates and extends several influential frameworks, including social exchange theory [39], social cognitive theory [32], and self-efficacy theory [40]. By combining these perspectives, we provide a more holistic understanding of the mechanisms through which FT, self-efficacy, and BL interact to influence job performance. This theoretical integration enriches the existing literature by offering a multidimensional perspective on the interplay of these critical factors.

From a practical standpoint, the findings present actionable recommendations for nursing management. Promoting a workplace culture that prioritizes trust and embraces BL aligns with contemporary calls for humanistic and employee-centered management practices in healthcare settings. Additionally, the study emphasizes the importance of interventions aimed at strengthening nurses' self-efficacy, which can lead to enhanced job performance, reduced turnover intention, and improved organizational outcomes. These findings are consistent with previous recommendations from Wang et al. [41] and Shahrour and Dardas [42], which advocate for leadership strategies that foster empowerment and trust within healthcare teams.

In conclusion, this study sheds light on the intricate relationships between FT, self-efficacy, BL, and job performance among nurses. By emphasizing the importance of fostering FT, cultivating self-efficacy, and encouraging BL, the study provides both theoretical insights and practical guidelines for enhancing nurse performance. These strategies hold significant potential to improve patient care quality and mitigate turnover intentions, thereby contributing to the overall success and sustainability of healthcare organizations.

## 6. Limitations and Future Research

This study provides valuable insights into the relationship between nurses' FT, self-efficacy, BL, and job performance. However, several limitations must be acknowledged. Firstly, the study relied on self-reported measures, which can be subject to bias and inaccuracies. To address this limitation, future studies could use more objective measures of these constructs. Secondly, the study adopted a cross-sectional design, which cannot establish causal relationships between the variables of interest. To overcome this limitation, future studies could adopt a longitudinal design or experimental designs to manipulate the variables of interest and test their causal effects on job performance.

Moreover, future studies could explore whether other variables, such as job satisfaction, burnout, or emotional exhaustion, mediate or moderate the relationships among FT, self-efficacy, BL, and job performance. Additionally, future research could examine the implications of the study's findings for patient outcomes. Although this study highlights the importance of creating a supportive organizational culture that fosters BL and promotes FT and self-efficacy among nurses, it does not directly examine the impact of these variables on patient care outcomes. Future research could address this gap by examining whether interventions aimed at promoting these variables have positive effects on patient satisfaction, safety, or clinical outcomes. By doing so, nursing management can develop evidence-based interventions that enhance the quality of patient care while improving job performance and reducing turnover intention.

## 7. Conclusion

In conclusion, this study offers important insights into the relationships between nurses' FT, self-efficacy, BL, and job performance. The findings indicate that nurses who experience higher levels of FT tend to achieve better job performance. Additionally, the study demonstrates that self-efficacy acts as a mediator in the relationship between FT and job performance, while BL serves as a moderator that enhances this connection. These results highlight the importance of cultivating a supportive organizational culture that promotes BL, strengthens FT, and enhances self-efficacy among nurses. By fostering such an environment, healthcare organizations can improve nurses' job performance, reduce turnover intentions, and elevate the quality of patient care. BL, characterized by genuine care for employees' well-being and development, plays a crucial role in creating an atmosphere of trust and empowerment. When combined with initiatives to boost self-efficacy, these efforts can significantly impact both individual and organizational outcomes. Overall, these findings carry critical clinical implications for nursing management. They provide a foundation for interventions designed to foster FT, self-efficacy, and BL, ultimately supporting nurses' performance and well-being while contributing to the delivery of higher-quality patient care.

## 8. Implications for Nursing Management

This study underscores the importance of FT in improving nurses' job performance, well-being, and overall commitment to patient care. Nursing managers play a pivotal role in fostering a trust-based work environment, where nurses feel recognized, valued, and empowered to take initiative. To achieve this, managers should adopt transparent communication practices, involve nurses in decision-making, and provide constructive feedback that reinforces their confidence and professional autonomy. By ensuring that nurses perceive trust from their supervisors, nursing leaders can enhance team cohesion, job satisfaction, and engagement, ultimately benefiting patient care outcomes.

The findings also highlight the mediating role of self-efficacy, suggesting that when nurses feel trusted, they develop a stronger belief in their capabilities, which translates into better performance. Nursing managers should invest in professional development programs, skills-based training, and mentorship initiatives that enhance nurses' confidence in handling complex clinical situations. Encouraging peer collaboration, cross-functional teamwork, and knowledge-sharing practices can further reinforce their self-efficacy, equipping them with the skills needed to adapt and thrive in demanding healthcare settings.

Furthermore, the moderating effect of BL suggests that a supportive and compassionate leadership style is essential in strengthening the relationship between FT and job performance. Nursing supervisors should demonstrate genuine care for nurses' well-being, acknowledge their contributions, and provide both emotional and professional support. Leadership training programs focusing on active listening, empathy, and mentorship can help develop these qualities among nurse leaders, ensuring that they foster a work culture where trust and collaboration flourish.

At the organizational level, hospital administrators should institutionalize trust-building mechanisms through policies that promote fairness, transparent workload distribution, and structured feedback systems. When nurses believe that their efforts are fairly recognized and that their concerns are addressed, they are more likely to stay engaged and contribute meaningfully to their teams. Additionally, organizations should implement well-being initiatives, flexible scheduling options, and burnout prevention strategies to create a healthy and sustainable nursing workforce.

## Figures and Tables

**Table 1:** Results of the confirmatory factor analysis of the variables in the study (*N* = 320).

Model	*χ* ^2^	df	*χ* ^2^/df	IFI	CFI	RMSEA
*Baseline model (four-factor model)*	779.04	301	2.59	0.97	0.96	0.07
*Three-factor model 1:* Benevolent leadership and job performance were combined into one factor	1889.75	304	6.22	0.88	0.88	0.13
*Three-factor model 2:* Benevolent leadership and self-efficacy were combined into one factor	2437.45	304	8.02	0.84	0.84	0.15
*Three-factor model 3:* Benevolent leadership and felt trust were combined into one factor	2126.09	304	6.99	0.87	0.87	0.14
*Three-factor model 4:* Job performance and self-efficacy were combined into one factor	1059.29	304	3.49	0.94	0.94	0.09
*Three-factor model 5:* Job performance and felt trust were combined into one factor	1565.29	304	5.15	0.91	0.91	0.11
*Three-factor model 6:* Self-efficacy and felt trust were combined into one factor	1934.20	304	6.36	0.88	0.88	0.13
*One-factor model*	3718.75	307	12.11	0.75	0.75	0.19

Abbreviations: CFI = comparative fit index, IFI = incremental fit index, RMSEA = root-mean-square error of approximation.

**Table 2:** Descriptions of demographic and job-related characteristics (*N* = 320).

Variables	Categories	Frequency	Percent (%)
Gender	Male	17	5.3
	Female	303	94.7

Age (years)	≤ 30	146	45.6
	31–40	152	47.5
	41–50	17	5.3
	≥ 51	5	1.6

Marital status	Single	92	28.7
	Married	220	68.8
	Others	8	2.5

Educational level	Junior college and below	58	18.1
	Bachelor's degree	252	78.8
	Master's degree and above	10	3.1

Annual income (thousand CNY)	< 50	44	13.8
	50–100	103	32.2
	110–150	110	34.4
	160–200	52	16.3
	210–250	11	3.4

Hospital level	Below Grade IIB	29	9.1
	Grade IIB	3	0.9
	Grade IIA	5	1.6
	Grade IIIB	26	8.1
	Grade IIIA	257	80.3

Work experience (years)	1–2	57	17.8
	3–5	70	21.9
	6–10	116	36.3
	11–15	43	13.4
	16–20	21	6.6
	> 21	13	4.1

Professional title	Nurse	49	15.3
	Nurse practitioner	192	60
	Nurse-in-charge	71	22.2
	Deputy chief nurse	8	2.5

**Table 3:** Means, standard deviations, and correlations of all variables in this study (*N* = 320).

Variables	Mean	SD	1	2	3	4	5	6	7	8	9	10	11	12
1. Gender	1.95	0.22	1											
2. Age	1.63	0.66	0.06	1										
3. Marital status	1.34	0.52	−0.03	−0.41^∗∗^	1									
4. Educational level	1.85	0.44	−0.05	0.06	−0.05	1								
5. Annual income	2.63	1.02	−0.13^∗^	0.25^∗∗^	−0.11^∗^	0.31^∗∗^	1							
6. Hospital level	4.50	1.20	−0.08	0.03	−0.02	0.46^∗∗^	0.47^∗∗^	1						
7. Work experience	2.81	1.29	0.04	0.67^∗∗^	−0.36^∗∗^	0.15^∗∗^	0.37^∗∗^	0.16^∗∗^	1					
8. Professional title	2.12	0.68	0.02	0.61^∗∗^	−0.38^∗∗^	0.27^∗∗^	0.28^∗∗^	0.17^∗∗^	0.60^∗∗^	1				
9. Felt trust	4.01	0.80	0.04	0.05	−0.06	0.11	0.04	0.19^∗∗^	0.10	0.07	1			
10. Benevolent leadership	3.82	0.86	0.00	−0.04	−0.04	0.13^∗^	0.04	0.22^∗∗^	0.07	0.00	0.69^∗∗^	1		
11. Self-efficacy	4.11	0.66	0.01	0.05	−0.05	0.11	0.10	0.21^∗∗^	0.11	0.08	0.75^∗∗^	0.61^∗∗^	1	
12. Job performance	4.22	0.62	0.02	0.04	−0.05	0.14^∗∗^	0.11^∗^	0.26^∗∗^	0.12^∗^	0.08	0.71^∗∗^	0.56^∗∗^	0.85^∗∗^	1

*Note:*;Gender: “1” = male, “2” = female; age: “1” = ≤ 30, “2” = 31–40, “3” = 41–50, “4” = ≥ 51; marital status: “1” = single, “2” = married, “3” = others; educational level: “1” = junior college and below, “2” = bachelor's degree, “3” = master's degree and above; annual income (thousand CNY): “1” =< 50, “2” = 50–100, “3” = 110–150, “4” = 160–200, “5” = 210–250; hospital level: “1” = below Grade IIB, “2” = Grade IIB, “3” = Grade IIA, “4” = Grade IIIB, “5” = Grade IIIA; work experience (years): “1” = 1-2, “2” = 3–5, “3” = 6–10, “4” = 11–15, “5” = 16–20, “6” = > 21; professional title: “1” = nurse, “2” = nurse practitioner, “3” = nurse-in-charge, “4” = ≥ deputy chief nurses.

^∗^ *p* < 0.05.

^∗∗^ *p* < 0.01.

**Table 4:** Results of hypothesis testing (*N* = 320).

Variables	Self-efficacy			Job performance		
	M1	M2	M3	M4	M5	M6
*Independent variable*						
Felt trust	0.62^∗∗^	0.52^∗∗^	0.54^∗∗^	0.54^∗∗^		0.13^∗∗^

*Mediator*						
Self-efficacy					0.77^∗∗^	0.66^∗∗^

*Moderator*						
Benevolent leadership		0.13^∗∗^	0.14^∗∗^			

*Interaction*						
FT × BL			0.07^∗∗^			
*F*	45.90^∗∗^	43.77^∗∗^	41.12^∗∗^	37.31^∗∗^	89.86^∗∗^	85.22^∗∗^
*R* ^2^	0.57	0.59	0.60	0.52	0.72	0.73
^△^ *R* ^2^	0.52	0.02	0.01	0.44	0.65	0.21

^∗∗^ *p* < 0.01.

**Table 5:** Results of bootstrap testing (*N* = 320).

Direct effect of *X* on *Y*	Effect	SE	LLCI	ULCI
FT ⟶ JB	0.13	0.04	0.06	0.19
Indirect effects of *X* in *Y*	Effect	BootSE	BootLLCI	BootULCI
FT ⟶ SE ⟶ JB	0.41	0.04	0.33	0.50

## Data Availability

The data that support the findings of this study are available from the corresponding author upon reasonable request.
